# Supplementary material for: SIRT1 Alleviates LPS-Induced IL-1β Production by Suppressing NLRP3 Inflammasome Activation and ROS Production in Trophoblasts
Source: Cells. 2020 Mar 16;9(3):728. doi: 10.3390/cells9030728 (PMC7140679; doi:10.3390/cells9030728)
Supplement: Supplementary file 1 [file cells-09-00728-s001.pdf]

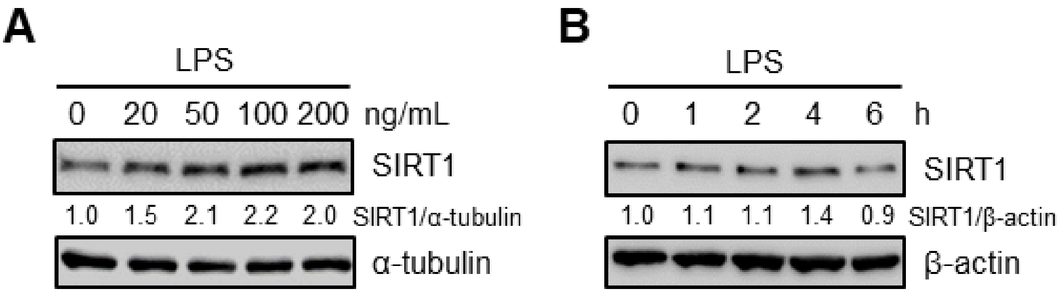

Figure S1. Expression of SIRT1 in HTR-8/SVneo cells exposed to LPS. (**A**) HTR-8/SVneo cells were treated with the indicated concentration of LPS for 4 h. Cell lysates were immunoblotted with an anti-SIRT1 antibody. α-Tubulin served as the loading control. Band intensities were quantified and normalized to the α-tubulin levels. (**B**) HTR-8/SVneo cells were treated with 200 ng/mL LPS for the indicated time. Cell lysates were immunoblotted with an anti-SIRT1 antibody. β-Actin served as the loading control. Numbers below the immunoblot bands indicate the fold changes normalized to the β-actin bands.

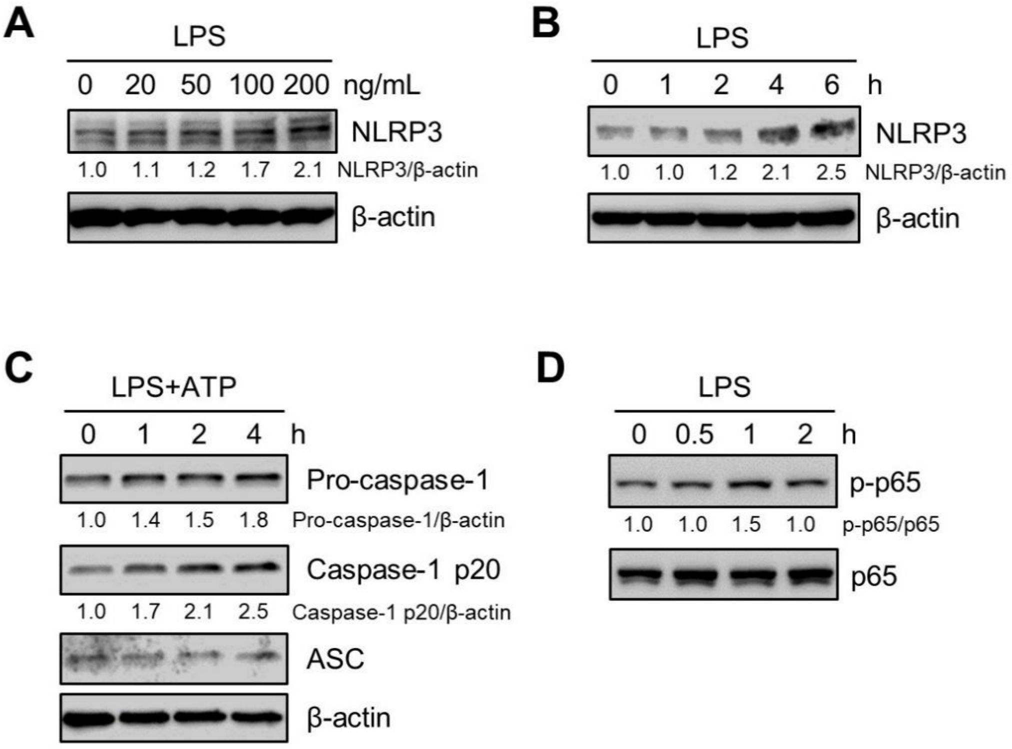

Figure S2. NLRP3 inflammasome activation in HTR-8/SVneo cells exposed to LPS. (**A**) HTR-8/SVneo cells were treated with the indicated concentration of LPS for 4 h. Cell lysates were immunoblotted with an anti-NLRP3 antibody. β-Actin served as the loading control. Numbers below the immunoblot bands indicate the fold changes normalized to the β-actin bands. (**B**,**D**) HTR-8/SVneo cells were treated with 200 ng/mL LPS for the indicated time. Cell lysates were immunoblotted with an anti-NLRP3, anti-p-p65 NF-κB, and anti-p65 NF-κB antibodies. (**C**) HTR-8/SVneo cells were treated with 200 ng/mL LPS for the indicated time and then followed by ATP (5 mM) treatment for 45 min. Cell lysates were immunoblotted with anti-Caspase-1 and anti-ASC antibodies. β-Actin or p65 served as the loading control. Numbers below the immunoblot bands indicate the fold changes normalized to the control bands.

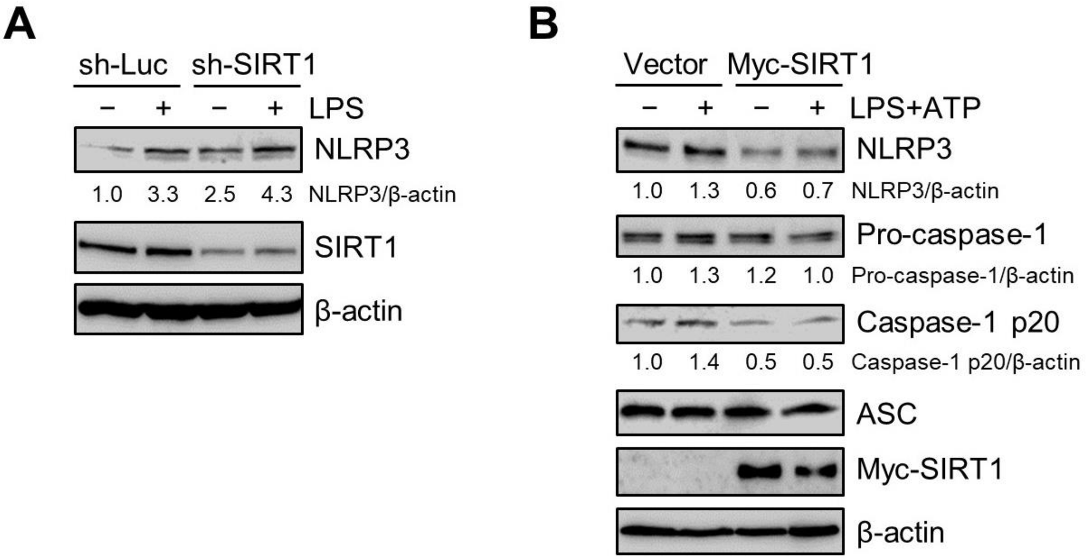

Figure S3. SIRT1 inhibits LPS-induced NLRP3 inflammasome activation (**A**) HTR-8/SVneo cells were infected with lentiviruses expressing shRNAs targeting luciferase (sh-Luc) or SIRT1 (sh-SIRT1) and then treated with 200 ng/mL LPS for 10 h. Cell lysates were immunoblotted with anti-NLRP3 and anti-SIRT1 antibodies. (**B**) Sw.71 cells were transfected with pcDNA3-myc-SIRT1 or pcDNA3 (empty vector) and treated with LPS (100 ng/mL) for 24 h and then followed by ATP (5 mM) treatment for 45 min, or were treated with vehicle. Cell lysates were immunoblotted with anti-NLRP3, anti-Caspase-1, anti-ASC, and anti-myc antibodies. β-Actin served as the loading control. Numbers below the immunoblot bands indicate fold changes normalized to the β-actin bands.

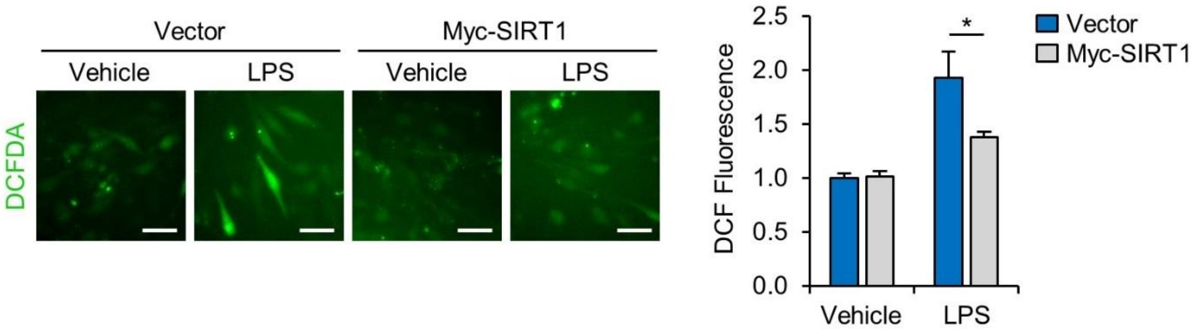

Figure S4. SIRT1 overexpression suppresses LPS-induced oxidative stress in Sw.71 cells. Sw.71 cells were transfected with pcDNA3-myc-SIRT1 or pcDNA3 (empty vector) and then treated with LPS (100 ng/mL) for 6 h. Reactive oxygen species (ROS) levels were analyzed using CM-H_2DCFDA. Scale bars, 50 μm. Data are indicated as mean ± SEM. Results are representative of at least three independent experiments. * $p < 0.05$ (Student's *t*-test).
